# Active-site solvent replenishment observed during human carbonic anhydrase II catalysis

**DOI:** 10.1107/S2052252517017626

**Published:** 2018-01-01

**Authors:** Jin Kyun Kim, Carrie L. Lomelino, Balendu Sankara Avvaru, Brian P. Mahon, Robert McKenna, SangYoun Park, Chae Un Kim

**Affiliations:** a Department of Physics, Ulsan National Institute of Science and Technology, Ulsan 44919, Republic of Korea; b Department of Biochemistry and Molecular Biology, College of Medicine, University of Florida, Gainesville, FL 32610, USA; c School of Systems Biomedical Science, Soongsil University, Seoul 06978, Republic of Korea

**Keywords:** carbonic anhydrase II, proton transfer, water dynamics, high-pressure cryocooling, active-site solvent replenishment

## Abstract

Human carbonic anhydrase II (hCA II) is a zinc metalloenzyme that catalyzes the reversible hydration/dehydration of CO_2_/HCO_3_^−^. Although hCA II has been extensively studied to investigate the proton-transfer process that occurs in the active site, its underlying mechanism is still not fully understood. Here, ultrahigh-resolution crystallographic structures of hCA II cryocooled under CO_2_ pressures of 7.0 and 2.5 atm are presented. The structures reveal new intermediate solvent states of hCA II that provide crystallographic snapshots during the restoration of the proton-transfer water network in the active site. Specifically, a new intermediate water (W_I_′) is observed next to the previously observed intermediate water W_I_, and they are both stabilized by the five water molecules at the entrance to the active site (the entrance conduit). Based on these structures, a water network-restructuring mechanism is proposed, which takes place at the active site after the nucleophilic attack of OH^−^ on CO_2_. This mechanism explains how the zinc-bound water (W_Zn_) and W1 are replenished, which are directly responsible for the reconnection of the His64-mediated proton-transfer water network. This study provides the first ‘physical’ glimpse of how a water reservoir flows into the hCA II active site during its catalytic activity.

## Abstract 2

Ultrahigh-resolution crystallographic structures of human carbonic anhydrase II (hCA II) cryocooled under CO_2_ pressures of 7.0 and 2.5 atm are presented. The structures reveal new intermediate solvent states of hCA II that provide crystallographic snapshots during restoration of the proton-transfer water network in the active site.

## 1. Introduction   

The reversible interconversion of carbon dioxide (CO_2_) and water to bicarbonate and a proton (H^+^) occurs at a rate that is limited by the diffusion of substrates in the presence of carbonic anhydrases (CAs) as the catalyst (Davenport, 1984 ▸; Christianson & Fierke, 1996 ▸; Chegwidden & Carter, 2000 ▸; Frost & McKenna, 2013 ▸; Supuran & De Simone, 2015 ▸). CAs are metalloenzymes that mostly contain zinc, although some are found with iron or cadmium. There are six distinct families of CA (α, β, γ, δ, ζ and the η family, which was recently subdivided from the α family) that are found throughout the animal, plant and bacterial kingdoms. In animals, CAs primarily function to maintain acid–base balance in the blood and other tissues, and to help the transport of CO_2_ out of tissues. In particular, mammalian CAs belong to the α family and are expressed as many different isozymes (Hewett-Emmett & Tashian, 1996 ▸). For instance, 14 forms of human α-CA can be divided into four cytosolic (I, II, III and VII), two mitochondrial (V_A_ and V_B_), one secreted (VI) and four membrane-bound (IV, IX, XII and XIV). The remaining three isoforms lack catalytic activity and are referred to as carbonic anhydrase-related proteins (CARPs). Among these isozymes, human CA II (hCA II) is expressed in most cell types, with involvement in many physiological processes (Krishnamurthy *et al.*, 2008 ▸; Frost & McKenna, 2013 ▸; Supuran & De Simone, 2015 ▸).

**Figure 1:**
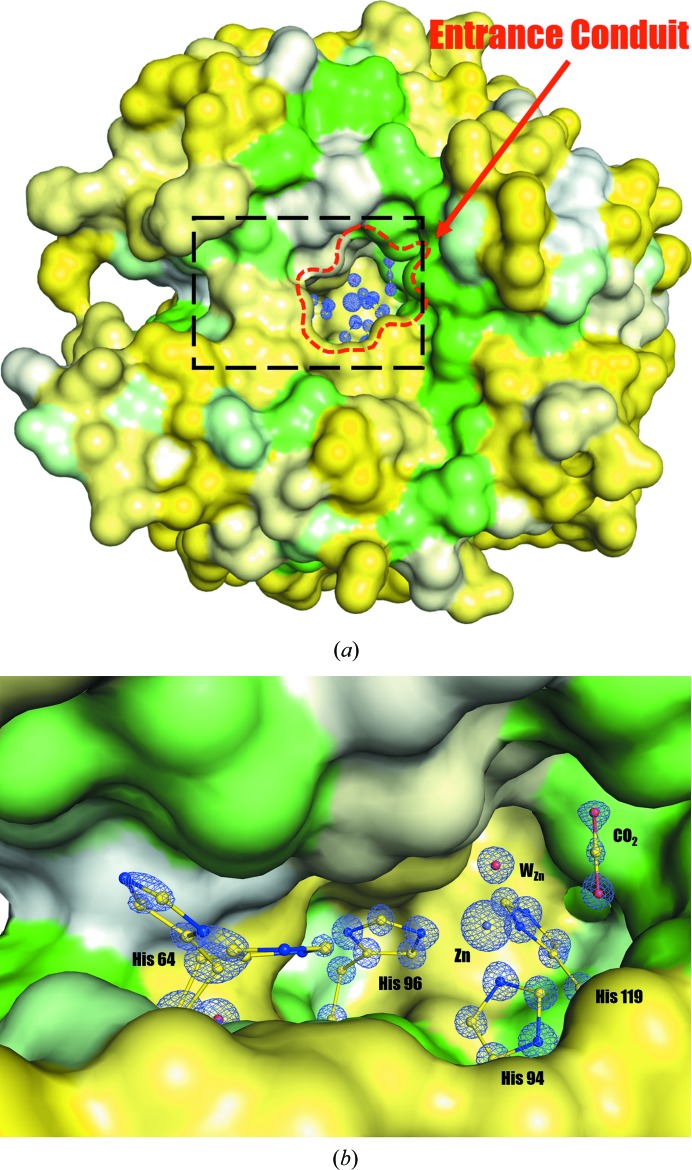
Surface rendition of hCA II depicted using the ultrahigh-resolution (0.9 Å) crystal structure of 7.0 atm CO_2_ hCA II. The hydrophobic and hydrophilic regions are coloured green and yellow, respectively. (*a*) The overall hCA II shows the CO_2_-binding active site that is located at a depth of 15 Å from the surface and is open to the outside bulk solvent through an entrance (the entrance diameter is ∼7–10 Å and it is referred to as the ‘entrance conduit’ in this study). The substrate and product (CO_2_/bicarbonate) of hCA II as well as water molecules can pass in and out through this open entrance conduit. (*b*) A closer view of the active site (Zn, CO_2_ binding site and His64) is shown through the entrance conduit with a 2*F*_o_ − *F*_c_ map (in blue) contoured at 2.5σ (for His64) and 5.0σ (others). The isolated electron density of the C atom of CO_2_ is clearly visible in this ultrahigh-resolution structure. The protein surface around His64 is removed and the proton-transfer water network (W_I_/W2/W3a/W3b) is not shown for clarity. The proton transfer during the catalytic activity is thought to occur *via* His64 through the proton wire rather than through the open entrance conduit.

The first crystal structure of hCA II, known at the time as hCA C, was determined by Liljas and coworkers in 1972 and was further refined in 1988 (Liljas *et al.*, 1972 ▸; Eriksson *et al.*, 1988 ▸). These studies laid the foundation for understanding the mechanism of CA activity. In hCA II, the active-site zinc is located within an ∼15 Å deep cleft and is tetrahedrally co­ordinated by three histidine residues (His94, His96 and His119) and an OH^−^ ion (Fig. 1 ▸). Furthermore, the active-site cavity subdivides into two distinct sides, formed by hydrophilic residues (*e.g.* Tyr7, Asn62, His64, Asn67, Thr199 and Thr200) and hydrophobic residues (*e.g.* Val121, Val143, Leu198, Thr199-CH_3_, Val207 and Trp209). The ‘hydrophobic’ side sequesters and positions the CO_2_ for nucleophilic attack by OH^−^ (Liang & Lipscomb, 1990 ▸).

Mechanistically, the conversion of CO_2_ to bicarbonate in the hydration direction takes place *via* the nucleophilic attack of CO_2_ by the zinc-bound hydroxide (OH^−^) (1). The subsequently generated bicarbonate is then displaced by a water molecule (W_Zn_; Silverman & Lindskog, 1988 ▸) (1):
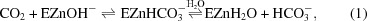


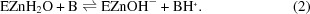



The next step of catalysis is the transfer of a proton from W_Zn_ to the bulk solvent, regenerating the zinc-bound OH^−^ (2). Here, the tetrahedral coordination of W_Zn_ to zinc causes polarization of the hydrogen–oxygen bond, making the O atom slightly more positive and thereby weakening the bond (Christianson & Fierke, 1996 ▸). The general base (B) for the proton transfer is likely to be mediated by ordered waters and His64 within the enzyme, where the hydrophilic side of the active site forms the hydrogen-bonded water network (W1, W2, W2′, W3a and W3b) that connects W_Zn_ to His64. This hydrogen-bonded network is believed to act as a proton wire that reduces the work required to transfer a proton from W_Zn_ to the bulk solvent for the regeneration of the zinc-bound OH^−^ (2) (Silverman & McKenna, 2007 ▸; Steiner *et al.*, 1975 ▸; Cui & Karplus, 2003 ▸; Fisher, Tu *et al.*, 2007 ▸; Zheng *et al.*, 2008 ▸; Fisher, Maupin *et al.*, 2007 ▸; Silverman *et al.*, 1979 ▸). Neutron studies have been utilized to observe the protonation states and orientation of water molecules in proteins (Langan *et al.*, 2008 ▸). Such experiments have determined that the water network in hCA II is pH-dependent, with an unbranched wire between W_Zn_ and His64 at physiological pH that is broken at high pH owing to a rearrangement of the hydrogen bonds of W1 (Budayova-Spano *et al.*, 2006 ▸; Fisher *et al.*, 2011 ▸). The side chain of His64 is oriented in two conformations, termed the ‘in’ (pointing towards the active site) and ‘out’ (pointing away from the active site) positions, that are suggested to facilitate the proton-shuttling process (Tu *et al.*, 1989 ▸; Fisher *et al.*, 2005 ▸; Nair & Christianson, 1991 ▸; Maupin & Voth, 2007 ▸; Lindskog, 1997 ▸; Avvaru *et al.*, 2010 ▸). Neutron structures have revealed that His64 is uncharged when occupying the ‘in’ position, priming the residue for the acceptance of a proton transferred from W_Zn_, regenerating the enzyme during catalysis (Fisher *et al.*, 2010 ▸). Moreover, the fact that binding of small molecules (activators) in the vicinity of His64 changes the catalytic rate by altering the proton-transfer step adds to the hypothesis that proton shuttling occurs *via* His64 (Supuran, 2008 ▸; Temperini *et al.*, 2005 ▸, 2006*a* ▸,*b* ▸; Briganti *et al.*, 1997 ▸, 1998 ▸).

Previously, the capture of CO_2_ in the active site of hCA II was achieved by cryocooling hCA II crystals under a 15 atm (1 atm = 101.325 kPa) CO_2_ pressure (Kim *et al.*, 2005 ▸; Domsic *et al.*, 2008 ▸). More recently, attempts have been made to track the intermediate changes during gradual CO_2_ release to the CO_2_-free state by incubating 15 atm CO_2_-pressurized hCA II crystals at room temperature (RT) for different time intervals (50 s, 3 min, 10 min, 25 min and 1 h) to decrease the internal CO_2_ pressure (Kim *et al.*, 2016 ▸). The resulting so-called intermediate snapshots revealed that two deep waters (W_DW_ and W′_DW_) immediately replace the vacated space as CO_2_ leaves the active site. In addition, W_I_ (intermediate water), which is only observed in fully CO_2_-bound hCA II (Domsic *et al.*, 2008 ▸), abruptly disappears, while W1 appears, as the CO_2_ is released. Moreover, with CO_2_ release W2′ (an alternate position of W2) in close proximity to residue His64 was observed to gradually disappear, whereas His64 concurrently rotated from the ‘out’ to the ‘in’ rotameric conformation. Despite the structural changes observed, the rapid changes taking place with the crystal incubation method left some of the key questions unanswered, such as how the proton-transfer water network is restored during hCA II catalytic activity.

In this study, we present ultrahigh-resolution structures of hCA II from crystals cryocooled under CO_2_ pressures of 7.0 atm (0.9 Å resolution) and 2.5 atm (1.0 Å resolution), which are hereafter referred to as ‘7.0 atm CO_2_ hCA II’ and ‘2.5 atm CO_2_ hCA II’, respectively. These two structures were compared with the three previous structures (Kim *et al.*, 2016 ▸) of hCA II crystals cryocooled under 15 atm CO_2_ pressure and then incubated at room temperature for 0 s (1.2 Å resolution structure from PDB entry 5dsi; hereafter referred to as ‘15 atm CO_2_ hCA II’), 50 s (1.25 Å resolution structure from PDB entry 5dsj; hereafter referred to as ‘15 atm CO_2_ hCA II – 50s’) and 1 h (1.45 Å resolution structure from PDB entry 5dsn; the ‘CO_2_-free state’ and hereafter referred to as ‘15 atm CO_2_ hCA II – 1h’). The structural comparison reveals that 7.0 atm CO_2_ hCA II and 2.5 atm CO_2_ hCA II are previously unknown intermediate states between 15 atm CO_2_ hCA II and 15 atm CO_2_ hCA II – 50 s. Together, these studies provide a view of how hCA II utilizes a water reservoir to fill the void in the active site as CO_2_ is released.

## 2. Experimental procedures   

### 2.1. Protein expression and purification   

The zinc-containing hCA II was expressed in a recombinant strain of *Escherichia coli* BL21 (DE3) pLysS transformed with a plasmid encoding the hCA II gene (Forsman *et al.*, 1988 ▸). Purification was carried out using affinity chromatography as described previously (Khalifah *et al.*, 1977 ▸). Briefly, bacterial cells were enzymatically lysed with hen egg-white lysozyme and the lysate was loaded onto agarose resin coupled with *p*-(aminomethyl)benzenesulfonamide, which binds to hCA II. The protein on the resin was eluted with 400 m*M* sodium azide in 100 m*M* Tris–HCl pH 7.0. The azide was removed by extensive buffer exchange against 10 m*M* Tris–HCl pH 8.0.

### 2.2. Protein crystallization   

Crystals of hCA II were obtained using hanging-drop vapour diffusion (McPherson, 1982 ▸). A 10 µl drop consisting of equal volumes of protein solution (5 µl) and well solution (5 µl) was equilibrated against 1 ml well solution (1.3 *M* sodium citrate, 100 m*M* Tris–HCl pH 7.8) at room temperature (∼20°C) (Domsic *et al.*, 2008 ▸). Crystals grew to approximate dimensions of 0.1 × 0.1 × 0.3 mm in a few days.

### 2.3. CO_2_ entrapment using pressure cryocooling   

CO_2_ entrapment was carried out as described in previous reports (Domsic *et al.*, 2008 ▸; Kim *et al.*, 2016 ▸). The hCA II crystals were first soaked in a cryosolution consisting of the reservoir solution supplemented with 20%(*v*/*v*) glycerol. The crystals were then coated with mineral oil to prevent dehydration and loaded into the base of high-pressure tubes. Once in the pressure tubes, the crystals were pressurized with CO_2_ gas to two different pressures (7.0 and 2.5 atm) at room temperature. After 10 min, the crystals were cryocooled to liquid-nitrogen temperature (77 K) without releasing the CO_2_ gas. Once the CO_2_-bound crystals had been fully cryocooled, the crystal-pressurizing CO_2_ gas was released and the crystal samples were stored in a liquid-nitrogen dewar until X-ray data collection. Note that once cryocooled, the CO_2_-bound hCA II crystals were handled just like normal protein crystals and were flash-cryocooled at ambient pressure.

### 2.4. X-ray diffraction and data collection   

Diffraction data were collected on CHESS beamline F1 (wavelength of 0.9180 Å, beam size of 100 µm) under a nitrogen cold stream (100 K). Data were collected using the oscillation method in intervals of 1° on an ADSC Quantum 270 CCD detector (Area Detector Systems Corporation) with a crystal-to-detector distance of 100 mm. For the 7.0 atm CO_2_ hCA II data set (0.9 Å resolution), an initial data set consisting of 180 images was collected with 1 s exposures to cover diffraction resolution up to 1.1 Å. The detector was then offset to cover diffraction resolution up to 0.88 Å, and a second data set consisting of 360 images was collected with 10 s exposures. For the 2.5 atm CO_2_ hCA II data set (1.0 Å resolution), a single data set consisting of 360 images was collected with 10 s exposure for each image. For each X-ray data set, the estimated absorbed X-ray dose was ∼2 × 10^7^ Gy. No significant diffraction resolution decay was observed up to this X-ray dose. Indexing, integration, merging and scaling were performed using *HKL*-2000 (Otwinowski & Minor, 1997 ▸). Data-processing statistics are given in Table 1 ▸.

### 2.5. Structure determination and model refinement   

The structures of hCA II at CO_2_ pressures of 7.0 and 2.5 atm were determined using the *CCP*4 program suite (Winn *et al.*, 2011 ▸). Prior to refinement, a random 5% of the data were flagged for *R*_free_ analysis. The previously determined 1.1 Å resolution crystal structure (PDB entry 3d92; Domsic *et al.*, 2008 ▸) was used as the initial phasing model. Maximum-likelihood refinement (MLH) was carried out using *REFMAC*5 (Murshudov *et al.*, 2011 ▸) and the water molecules were automatically picked up using *ARP*/*wARP* (Perrakis *et al.*, 1999 ▸) during the MLH cycles. The refined structures were manually checked using the molecular graphics program *Coot* (Emsley & Cowtan, 2004 ▸). Reiterations of MLH refinement were carried out with anisotropic *B* factors and riding H atoms. The partial occupancies of W1 in 7.0 and 2.5 atm CO_2_ hCA II were estimated such that the electron density in the *F*_o_ − *F*_c_ map disappears. The refinement statistics are given in Table 1 ▸. We also re-refined the water molecules in the three previously reported structures (PDB entry 5dsi for 15 atm CO_2_ hCA II, PDB entry 5dsj for 15 atm CO_2_ hCA II – 50s and PDB entry 5dsn for CO_2_ hCA II – 1h; Kim *et al.*, 2016 ▸) for accurate comparison of the bound water molecules in the active site and the entrance conduit. The re-refined structures were updated in the PDB with the new PDB codes 5yui (superseding 5dsi), 5yuj (superseding 5dsj) and 5yuk (superseding 5dsn). Details of the structural analysis of the bound water molecules are given in the Supporting Information. All structural figures were rendered with *PyMOL* (Schrödinger).

## 3. Results and discussion   

### 3.1. CO_2_ binding sites: active site (CO_2_/W_Zn_/W_DW_/W′_DW_) and secondary CO_2_ site near Phe226   

**Figure 2:**
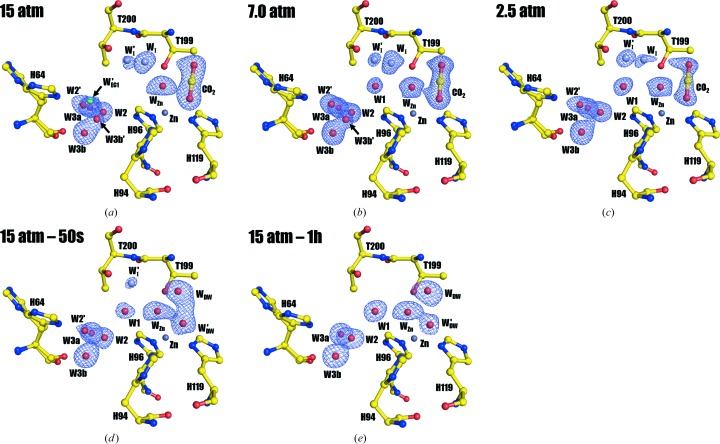
The active site of hCA II at different internal CO_2_ pressures. (*a*) 15 atm CO_2_ hCA II, (*b*) 7.0 atm CO_2_ hCA II, (*c*) 2.5 atm CO_2_ hCA II, (*d*) 15 atm CO_2_ hCA II – 50s, (*e*) 15 atm CO_2_ hCA II – 1h. The electron-density (2*F*_o_ − *F*_c_) map (in blue) is contoured at 1.3σ, except for W′_I_ in (*d*), which is contoured at 1.0σ. The intermediate waters W_I_ and W′_I_ are coloured light grey and the entrance-conduit water W′_EC1_ is coloured cyan for clarity. Note that CO_2_ is fully bound in the active site in (*a*) and (*b*). Concurrent with the decrease in CO_2_ pressure, the electron density for CO_2_ fades out in (*c*) and is eventually replaced by two water molecules in the CO_2_ binding site (*d*, *e*). Note also the dynamic changes reflected by the electron densities of W_I_, W′_I_ and W1 that take place as the internal CO_2_ pressure decreases. These events are more explicitly explained in Fig. 4 ▸.

Structural examinations show that the five structures are very similar. The all-protein-atom r.m.s.d.s between 15 atm CO_2_ hCA II and the other four structures (7.0 atm CO_2_ hCA II, 2.5 atm CO_2_ hCA II, 15 atm CO_2_ hCA II – 50s and 15 atm CO_2_ hCA II – 1h) are 0.14, 0.12, 0.10 and 0.13 Å, respectively. Although changes in the zinc-coordinating histidines (His94, His96 and His119) are negligible between the structures, the electron densities for the CO_2_ binding site differ significantly, as expected (Fig. 2 ▸). While 15 and 7.0 atm CO_2_ hCA II show a clear position for the CO_2_ (Figs. 2 ▸ *a* and 2 ▸ *b*), deterioration of electron density for the CO_2_ site occurs in the 2.5 atm CO_2_ hCA II structure, represented by sparsely connected lobes (Fig. 2 ▸ *c*). When the density is modelled and refined with only CO_2_, the CO_2_ occupancy is at most 0.7. This difference suggests that the CO_2_ site is occupied by both CO_2_ and two waters (deep waters W_DW_ and W′_DW_) at a pressure of 2.5 atm. The manifestation of W_DW_ at this pressure is supported by the extended electron-density connection from CO_2_ to W_I_ (Supplementary Fig. S1). In 15 atm CO_2_ hCA II – 50s, the electron density for the CO_2_ binding site is further shifted towards Zn and W_Zn_, which correlates with the known positions of W_DW_ and W′_DW_ (Fig. 2 ▸ *d*). This argues that 2.5 atm CO_2_ hCA II has a higher internal CO_2_ pressure than 15 atm CO_2_ hCA II – 50s. Finally, in 15 atm CO_2_ hCA II – 1h, the electron density of the CO_2_ binding site splits into two distinct lobes, indicating that the CO_2_ site is completely replaced by W_DW_ and W′_DW_ (Fig. 2 ▸ *e*).

Previously, the binding of a secondary CO_2_ molecule which is 15–16 Å away from the active-site CO_2_ molecule was reported in a hydrophobic pocket created by Val223 and Phe226 (Domsic *et al.*, 2008 ▸). Comparison of the 15 atm CO_2_ hCA II and 15 atm CO_2_ hCA II – 1h structures in this region indicates that the side chain of Phe226 must rotate to accommodate the secondary CO_2_ molecule (Supplementary Figs. S2*a* and S2*e*). Interestingly, in the lower pressured 7.0 atm CO_2_ hCA II, the subdued electron density for the secondary CO_2_ results in dual conformations of the Phe226 side chain (Supplementary Fig. S2*b*). In the cases of 2.5 atm CO_2_ hCA II and 15 atm CO_2_ hCA II – 50s, the secondary CO_2_ was not present and the Phe226 side chain sits in the position observed in the CO_2_-free 15 atm CO_2_ hCA II – 1h (Supplementary Figs. S2*c*, S2*d* and S2*e*). Hence, the observation of the secondary CO_2_ and the dual conformations of the Phe226 side chain in 7.0 atm CO_2_ hCA II imply that 7.0 atm CO_2_ hCA II has a higher internal CO_2_ pressure than 15 atm CO_2_ hCA II – 50s.

### 3.2. His64 and the water network (W1/W_I_/W2/W2′/W3/W3a/W3b) near the active site   

**Figure 3:**
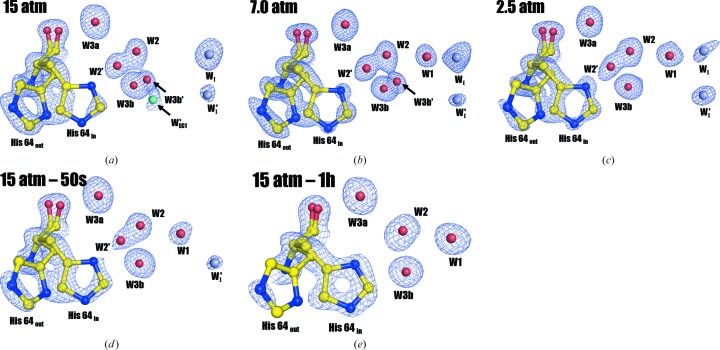
Rotameric states of His64 and solvent positions at different internal CO_2_ pressures. (*a*) 15 atm CO_2_ hCA II, (*b*) 7.0 atm CO_2_ hCA II, (*c*) 2.5 atm CO_2_ hCA II, (*d*) 15 atm CO_2_ hCA II – 50s, (*e*) 15 atm CO_2_ hCA II – 1h. The electron density (2*F*_o_ − *F*_c_) for W′_I_ in (*d*) is contoured at 1.0σ. In all other cases, the electron density (2*F*_o_ − *F*_c_) for His64 and the electron density (2*F*_o_ − *F*_c_) for waters are contoured at 1.5 and 1.3σ, respectively. The intermediate waters W_I_ and W′_I_ are coloured light grey and the entrance-conduit water W′_EC1_ is coloured cyan for clarity. As the internal CO_2_ pressure decreases, W2′ gradually dissipates and the His64 side chain shifts from the ‘out’ to the ‘in’ position from (*a*) to (*e*). The intermediate water, W_I_, is clearly observed in (*a*) and the electron density gradually subsides (*b*, *c*) and finally disappears (*d*, *e*). In accordance to the decrease in W_I_, electron density for W1, which is not observable in (*a*), appears in (*b*) and subsequently increases gradually (*c*, *d*, *e*). When the models are refined with partial occupancy, the W1 occupancies are 0.8 in (*b*) and 0.9 in (*c*). Interestingly, the electron density for the newly observed intermediate water W′_I_ increases gradually from (*a*) to (*c*), but decreases in (*d*) and disappears in (*e*). The measured distance between W_I_ and W1 in (*b*) and (*c*) is 2.0 Å. The electron density for W3a is well isolated in all cases, but W3b shows an alternate position W3b′ in (*a*) which grows in (*b*) but subsequently disappears (*c*, *d*, *e*).

As described above, structural examinations of the CO_2_ binding sites suggest that both 7.0 atm CO_2_ hCA II and 2.5 atm CO_2_ hCA II have a higher internal CO_2_ pressure than 15 atm CO_2_ hCA II – 50 s. Furthermore, 7.0 atm CO_2_ hCA II intuitively has a higher internal CO_2_ pressure than 2.5 atm CO_2_ hCA II, hence leading to the conclusion that the internal CO_2_ pressure decreases in the sequence 15 atm CO_2_ hCA II, 7.0 atm CO_2_ hCA II, 2.5 atm CO_2_ hCA II, 15 atm CO_2_ hCA II – 50s, 15 atm CO_2_ hCA II – 1h. Such an interpretation ascertains that 7.0 atm CO_2_ hCA II and 2.5 atm CO_2_ hCA II are intermediate states that fill the gaps between the 15 atm pressurized CO_2_ hCA II and the earliest time point of CO_2_ release (15 atm CO_2_ hCA II – 50s) observed in the previous study (Kim *et al.*, 2016 ▸). On this foundation, His64 and the water network near the active site were analyzed in order of decreasing internal CO_2_ pressure (Fig. 3 ▸).

Although the side chain of His64 lies predominantly in the ‘out’ position in 15 atm CO_2_ hCA II (Fig. 3 ▸ *a*), the electron density of His64 infers that it occupies dual ‘out’ and ‘in’ positions as the internal CO_2_ pressure decreases (Figs. 3 ▸ *b*, 3 ▸ *c* and 3 ▸ *d*). However, in the CO_2_-free 15 atm CO_2_ hCA II – 1h, His64 is observed to primarily occupy the ‘in’ position (Fig. 3 ▸ *e*). In concert with His64 moving from the ‘out’ to the ‘in’ position, the density for W2′ (an alternate position of W2) is observed to gradually dissipate.

In the previous studies, it has been recognized that when CO_2_ is fully bound in the 15 atm CO_2_ hCA II structure, W_I_ but not W1 is observed (as in Figs. 2 ▸ *a* and 3 ▸ *a*; Kim *et al.*, 2016 ▸). However, this W_I_ disappeared in 15 atm CO_2_ hCA II and W1 was observed to appear instead in the CO_2_-free 15 atm CO_2_ hCA II – 1h (as in Figs. 2 ▸ *e* and 3 ▸ *e*; Kim *et al.*, 2016 ▸). Because the measured distance between W_I_ and W2 is ∼4.8 Å, the hydrogen-bonded water network (*via* W1, W2 and His64) necessary for the proton-transfer wire was presumed to be broken when CO_2_ fully binds to the active site. In this study, we observed the dynamic replacement of W_I_ with W1 as the internal CO_2_ pressure decreases, since dually occupied positions of W1 and W_I_ are seen for 7.0 and 2.5 atm CO_2_ hCA II (Figs. 3 ▸ *b* and 3 ▸ *c*). The reduction of electron density for W_I_ is observed in the order 15, 7.0 and 2.5 atm CO_2_ hCA II, with complete disappearance in 15 atm CO_2_ hCA II – 50s and 15 atm CO_2_ hCA II – 1h (Fig. 3 ▸). In contrast, W1 electron density starts to emerge in the 7.0 atm CO_2_ hCA II, is more pronounced in 2.5 atm CO_2_ hCA II, and is fully occupied in 15 atm CO_2_ hCA II – 50s and 15 atm CO_2_ hCA II – 1h. The close 2.0 Å distance between W1 and W_I_ in 7.0 and 2.5 atm CO_2_ hCA II suggests that W1 and W_I_ exhibit partial occupancies rather than being two separate, stable waters. The inverse correlation, with a decrease in W_I_ and increase in W1 electron density upon decreasing internal CO_2_ pressures, suggests that W_I_ moves to the W1 position upon CO_2_ release.

### 3.3. New observations of alternate W_I_ (W′_I_), alternate W3b (W3b′) and entrance-conduit waters (W_EC1_/W_EC2_/W_EC3_/W_EC4_/W_EC5_)   

This study reveals newly observed features in the water network within and at the entrance to the hCA II active site. Along with the previously reported intermediate water W_I_, another well ordered intermediate water W′_I_ (in this study) was observed in the structures of 7.0 and 2.5 atm CO_2_ hCA II (Figs. 2 ▸, 3 ▸ *b* and 3 ▸ *c*). When the previously reported structures were compared, W′_I_ existed in the 15 atm CO_2_ hCA II and 15 atm CO_2_ hCA II – 50s structures, but was overlooked because of its faint electron density (Figs. 2 ▸, 3 ▸ *a* and 3 ▸ *d*). Compared with W_I_, W′_I_ is located farther away from the active site and more towards the entrance that connects the active site of hCA II to bulk solvent. Because the entrance is near the active site where water, substrate and product (CO_2_/bicarbon­ate) can interchange/interact with bulk solvent, we will refer to it as the ‘entrance conduit’ (Fig. 1 ▸). The conduit consists of the hydrophobic residues Leu198, Val135, Leu204, Pro202 and Phe131 on one side, and the hydrophilic residues His64, Gln92 and Thr200 on the other. It should also be noted that the proton-shuttling His64 is positioned perpendicularly to this entrance conduit (Fig. 1 ▸).

**Figure 4:**
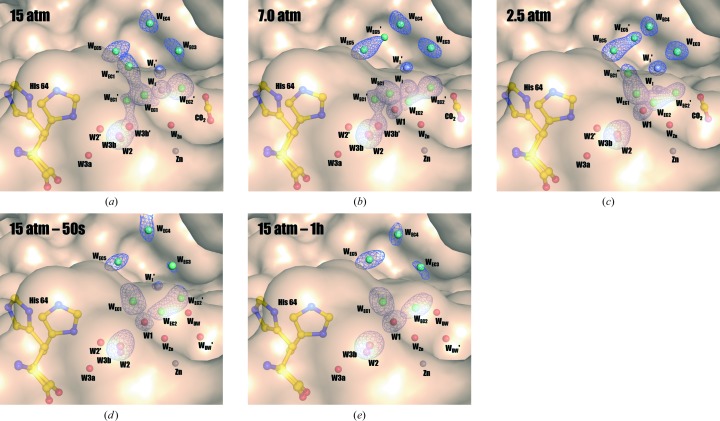
Solvent positions in the entrance conduit. (*a*) 15 atm CO_2_ hCA II, (*b*) 7.0 atm CO_2_ hCA II, (*c*) 2.5 atm CO_2_ hCA II, (*d*) 15 atm CO_2_ hCA II – 50s, (*e*) 15 atm CO_2_ hCA II – 1h. In all cases the electron-density (2*F*_o_ − *F*_c_) maps are contoured at 1.3σ, except for the electron density (2*F*_o_ − *F*_c_) for W′_I_ in (*d*), which is contoured at 1.0σ. The intermediate waters W_I_ and W′_I_ are coloured light grey and the entrance-conduit waters are coloured cyan for clarity. As the internal CO_2_ pressure decreases, alternate positions appear and disappear, especially for W_EC1_, W_EC2_ and W_EC5_, suggesting dynamical motions that are correlated with the dynamical changes in W_I_, W′_I_ and W1 (*a*, *b*, *c*). For example, note that W′_EC1_ in (*a*) and (*b*) is located next to the electron density for W3b′, attesting to the interaction between the two. As W_I_ and W′_I_ disappear in (*d*) and (*e*) together with appearance of W1, the entrance-conduit water molecules return to the singly ordered positions (*d*, *e*).

A close inspection of the structures further identified five water molecules (named entrance-conduit waters or W_EC_s) that are ordered along the surface of the entrance conduit in the CO_2_-free 15 atm CO_2_ hCA II – 1h (sequentially named counterclockwise as W_EC1_, W_EC2_, W_EC3_, W_EC4_ and W_EC5_ starting from the one closest to water W3b; Fig. 4 ▸). Unlike for W_EC3_ and W_EC4_, alternate positions for W_EC1_ (W′_EC1_ and W′′_EC1_), W_EC2_ (W′_EC2_) and W_EC5_ (W′_EC5_) exist in the different internal CO_2_ pressure structures. Tight hydrogen-bonding networks stabilize W_EC1_, W_EC2_, W_EC3_, W_EC4_ and W_EC5_, which are mediated by residues lining the entrance conduit (Supplementary Fig. S3). For instance, the side-chain amide N atom of Gln92 binds to W_EC2_, and the main-chain carbonyl O atom of Pro201 and the side-chain hydroxyl O atom of Thr200 bind to W_EC5_, which are conserved throughout all of the internal CO_2_ pressure structures. Hydrogen-bonding inter­actions also exist in all of the structures between the five W_EC_ waters (W_EC1_–W_EC2_, W_EC2_–W_EC3_, W_EC3_–W_EC4_ and W_EC4_–W_EC5_).

The intermediate waters W_I_ and W′_I_ are located deep within this conduit near the active site and several W_EC_ waters are involved in transiently stabilizing them and water W1 (Supplementary Fig. S4). Among the W_EC_ waters, W_EC2_, W_EC3_ and W_EC5_ participate in stabilizing W_I_, W′_I_ and W1 within all of the structures. For example, hydrogen-bonding interactions with W′_I_ are observed for W_EC2_, W_EC3_ and W_EC5_, while hydrogen-bonding interactions with W_I_ and W1 are observed for W_EC2_.

**Figure 5:**
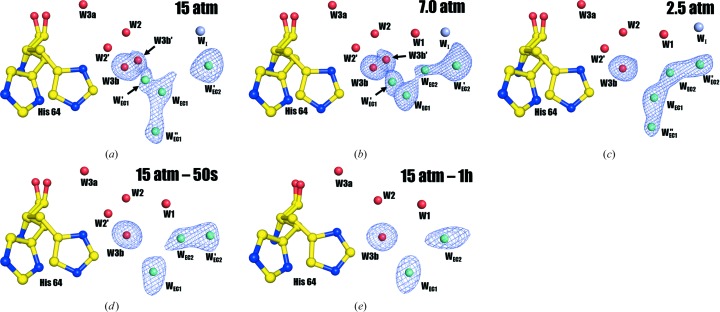
Entrance-conduit water dynamics. (*a*) 15 atm CO_2_ hCA II, (*b*) 7.0 atm CO_2_ hCA II, (*c*) 2.5 atm CO_2_ hCA II, (*d*) 15 atm CO_2_ hCA II – 50s, (*e*) 15 atm CO_2_ hCA II – 1h. In all cases, the electron-density (2*F*_o_ − *F*_c_) maps are contoured at 1.3σ. The intermediate water W_I_ is coloured light grey and the entrance-conduit waters are coloured cyan for clarity. Although all five W_EC_ waters (W_EC1_, W_EC2_, W_EC3_, W_EC4_ and W_EC5_) exist in all of the structures regardless of the different internal CO_2_ pressures, dramatic variations of W_EC1_ (near to the proton-shuttling His64), W_EC2_ (near to W_I_, W′_I_ and W1) and W3b′ are manifested by multiple alternate positions during the internal CO_2_ pressure decrease. These observations indicate that the waters of the proton-transfer network (W1/W2/W2′/W3a/W3b/W3b′), the intermediate waters (W_I_/W′_I_) and the entrance conduit waters (W_EC1_/W_EC2_/W_EC3_/W_EC4_/W_EC5_) all act interdependently and are dynamically correlated.

Although all five W_EC_ waters are present regardless of the different internal CO_2_ pressures, some perturbations of W_EC1_ (near to the proton-shuttling His64) and W_EC2_ (near to W_I_, W′_I_ and W1) were observed during the internal CO_2_ pressure decrease, which are manifested by multiple alternate positions (Fig. 5 ▸). The dynamic motions of W_EC_ waters imply their direct interplay with the proton-transfer water network in the active site. Specifically, interactions between W_EC1_ and W3b were observed. Previously, the positions of W3a and W3b were thought to be invariant and singly occupied regardless of CO_2_ binding, leading to the belief that the main role of W3a and W3b was to stabilize the W2 water molecule that is directly located within the proton-transfer wire. However, in this study an alternate position of W3b [named W3b′, which is different from the two alternative positions (W3b′ and W3b′′) in CO_2_-bound apo CA II in Kim *et al.* (2016 ▸)] was observed along with an alternate position of W_EC1_ (named W′_EC1_ in this study) in 15 and 7.0 atm CO_2_ hCA II (Figs. 5 ▸ *a* and  ▸ *b*). In these structures, the distances between W3b, W3b′, W_EC1_ and W′_EC1_ are so close (1.3–1.7 Å; Supplementary Fig. S5) that they organize into a continuous tube of electron density (Fig. 5 ▸). W3b′ and W′_EC1_ disappear in lower internal CO_2_ pressure structures, with W_EC1_ recovering to the singly occupied position (Figs. 5 ▸c, 5 ▸ *d* and 5 ▸ *e*). These results suggest that the waters in the proton-transfer network (W1/W2/W2′/W3a/W3b/W3b′), the intermediate waters W′_I_/W_I_ and the water network of the entrance conduit (W_EC1_/W_EC2_/W_EC3_/W_EC4_/W_EC5_) all act interdependently with their motions correlated.

### 3.4. Mechanism of the restoration of the proton-transfer water network   

By lowering the CO_2_ pressure in hCA II crystals, we captured additional intermediate states, including dual occupied positions of W1 and W_I_, an active site partially occupied with CO_2_, W_DW_ and W′_DW_, a new intermediate water W′_I_ and an alternate position of W3b (W3b′). By realizing that W_I_ and W′_I_ are transiently stabilized by several entrance-conduit water molecules and that they rearrange during the restoration of the proton-transfer water network, we propose the sequential events in the formation of the water network that replenishes W_Zn_ and the consequential connection of the His64-mediated proton-transfer wire during the catalytic turnover of hCA II. Although these events have been postulated from the observations during CO_2_ release in this study, these mechanisms may also account for restoration of the water network after bicarbonate release, assuming that both CO_2_ and bicarbonate molecules do not directly mediate the water-network restoration process.

**Figure 6:**
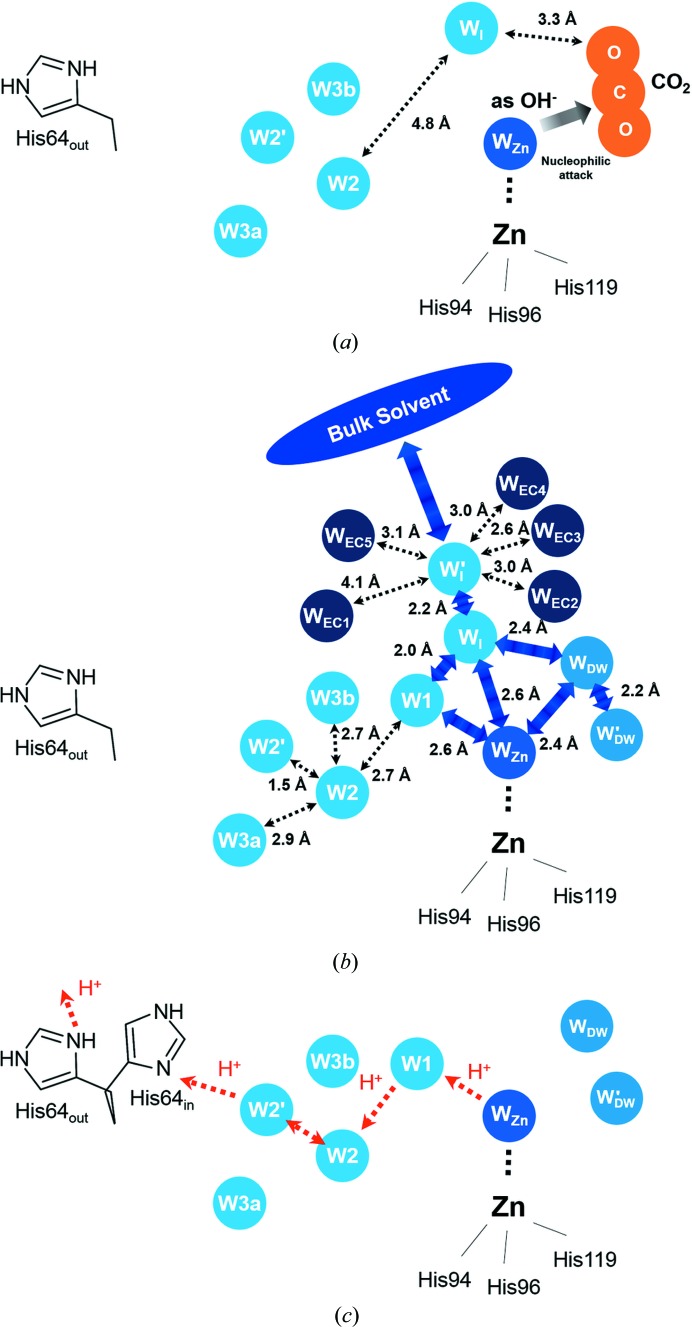
Proposed mechanism of water-network restoration. (*a*) In hCA II with fully bound CO_2_, W_Zn_ exists as OH^−^ and nucleophilic attack occurs to form bicarbonate. In this state, only W_I_ is present and not W1, suggesting that the water network for proton transfer is broken. (*b*) The sequential events for water-network restoration as the product bicarbonate leaves the active site. After the intermediate water W_I_ fills the places for W1 and W_DW_, and these W1 and W_DW_ waters or W_I_ can move to the W_Zn_ position. W_DW_ also fills the position for W′_DW_. Note that four waters (W1, W_Zn_, W_DW_ and W′_DW_) are eventually filled in from W_I_ during this water-network restoration process. A newly found intermediate water W′_I_ is located between W_I_ and the outside bulk solvent, is stabilized by the entrance-conduit waters and seems to facilitate the fast charging process of W_I_. (*c*) Only after the water network is restored can proton transfer can occur from W_Zn_ to the outside through W1/W2/W2′/His64_in_/His64_out_. Now, with CO_2_ binding, hCA II is ready for the next catalytic turnover.

It is observed that only W_I_, and not W1, is observed near the active site of hCA II in the fully CO_2_-bound state (Fig. 6 ▸ *a*). Because the distance between W_I_ and W2 is ∼4.8 Å, the lack of W1 suggests that the hydrogen-bonded water network from W_Zn_ to His64 (charged and in the ‘out’ position) is disrupted. In fact, when hCA II is fully CO_2_-bound, the proton transfer should have already happened to result in the deprotonation of W_Zn_ to OH^−^, which is necessary for the nucleophilic CO_2_ attack (the first step in equation 1). After bicarbonate formation *via* this nucleophilic attack, the product bicarbonate subsequently leaves the active site and W_Zn_ is replenished along with restoration of the active-site water network prior to the proton-transfer process from W_Zn_.

It is likely that W_Zn_ replenishment and water-network restoration are directly mediated by the transient waters W_I_ and W′_I_. After bicarbonate diffuses out of hCA II, W_I_ seems to immediately fill the positions of both W1 and W_DW_ (Fig. 6 ▸ *b*). This directional branching movement of W_I_ is predictable since the distance between W_I_ and W1 is 2.0 Å and the distance between W_I_ and W_DW_ is 2.4 Å. This interpretation is further supported by the observation that the electron density of W1 emerges as that of W_I_ disappears (Figs. 3 ▸ *a*, 3 ▸ *b*, 3 ▸ *c* and 3 ▸ *d*), as well as by the observation that the electron density of W_I_ is fused to the electron density of W_DW_ (Supplementary Fig. S1). Subsequently, W1 can move to W_Zn_ (the distance between W1 and W_Zn_ is 2.6 Å), and W_DW_ can shift to either W_Zn_ or W′_DW_ (the distances from W_DW_ to W_Zn_ and from W′_DW_ are 2.4 and 2.2 Å, respectively). Judging by the distance from W_I_ to W_Zn_ (2.6 Å), W_I_ can also directly flow into the W_Zn_ position (Fig. 6 ▸ *b*).

As W_I_ replenishes multiple water positions (W1/W_DW_/W′_DW_/W_Zn_), it is important that the bulk solvent supplies the W_I_ position rapidly (acting as a water reservoir), a process that seems to be facilitated by W′_I_. W′_I_ is separated from W_I_ by 2.2 Å, is located closer to the bulk solvent and is transiently stabilized by the dynamic motions of water molecules in the entrance conduit, which take place in concert with the changes of solvent in the active site (Fig. 4 ▸ and Supplementary Fig. S4). As the W1/W_DW_/W′_DW_/W_Zn_ positions are filled, the intermediate water W_I_ is destabilized by steric hindrance with W1 (the distance between W1 and W_I_ is only 2.0 Å). In addition, the dynamic motions of water molecules in the entrance conduit decrease as the water network is restored in the active site (Fig. 5 ▸), which causes destabilization of W′_I_. Therefore, the intermediate waters W_I_ and W′_I_ disappear. Finally, the active-site water network is fully restored and proton transfer occurs from W_Zn_ to His64 (uncharged and in the ‘in’ position) *via* W1/W2/W2′ (Fig. 6 ▸ *c*).

## 4. Conclusions   

Structural comparisons between hCA II in complex with CO_2_ and during its release reveal intermediate snapshots during the water-network rearrangement in the active site as the waters fill the void following CO_2_ liberation. Based on our observations, insight into the water-network restoration prior to proton transfer is proposed. While previous studies of the catalytic activity of hCA II have mainly focusing on the CO_2_ binding site (Zn/W_Zn_/W_DW_) and the proton-transfer water network (W1/W2/W3a/W3b), our results indicate that the intermediate and alternate waters (W′_I_, W_I_, W2′ and W3b′) and the entrance-conduit waters (W_EC1_, W_EC2_, W_EC3_, W_EC4_, W_EC5_ and their alternative positions) are critically involved in catalysis by hCA II. The substrate CO_2_ enters *via* the hydrophobic half of the active site, while the product HCO_3_^−^, being a charged molecule, exits by perturbing the ordered waters that fill the hydrophilic half of the active site (Silverman *et al.*, 1979 ▸; Koenig *et al.*, 1983 ▸). Thus, the ordered waters within the active site and its vicinity are likely to exist in a state of intermittent rearrangement during the forward and reverse reactions of catalysis. Taken collectively, our results provide snapshots of low-energy stages of water rearrangement during catalysis. Future mutation studies to perturb the protein regions that stabilize these waters would provide more evidence of their roles in the reaction. Moreover, our results suggest that the catalytic activity of hCA II can be more thoroughly understood with the ‘extended’ catalytic waters (W_DW_/W′_DW_/W_Zn_/W1/W_I_/W′_I_/W2/W2′/W3a/W3b/W3b′/W_EC1_–W_EC5_). Molecular dynamics simulations on this extended water network may reveal further insights into the bioenergetic mechanisms utilized by hCA II to generate ordered water networks from the surrounding disordered bulk solvent (Riccardi *et al.*, 2006 ▸; Roy & Taraphder, 2007 ▸).

## Supplementary Material

PDB reference: 2.5 atm CO_2_ hCA II, 5y2r

PDB reference: 7 atm CO_2_ hCA II, 5y2s

PDB reference: 15 atm CO_2_ hCA II, re-refined, 5yui

PDB reference: 15 atm CO_2_ hCA II – 50s, re-refined, 5yuj

PDB reference: 15 atm CO_2_ hCA II – 1h, re-refined, 5yuk

Supplementary information, figures and table.. DOI: 10.1107/S2052252517017626/mf5021sup1.pdf

## Figures and Tables

**Table 1:** Data-collection and refinement statistics for 7.0 and 2.5 atm CO_2_ hCA II Values in parentheses are for the highest resolution shell.

CO_2_ pressure	7.0 atm	2.5 atm
Data collection		
Space group	*P*2_1_	*P*2_1_
*a*, *b*, *c* (Å)	42.31, 41.37, 71.94	42.28, 41.41, 72.11
α, β, γ (°)	90, 104.12, 90	90, 104.16, 90
Resolution (Å)	30–0.90 (0.92–0.90)	30–1.00 (1.02–1.00)
*R*_merge_ (%)	12.1 (42.6)	9.1 (56.4)
〈*I*/σ(*I*)〉	14.1 (1.9)	25.1 (3.0)
Completeness (%)	95.2 (73.6)	98.6 (96.3)
Multiplicity	4.7 (2.8)	7.1 (5.2)
Refinement		
Resolution (Å)	0.9	1.0
No. of reflections	162430	122146
*R*_work_/*R*_free_ (%)	11.1/12.7	11.4/13.1
No. of atoms		
Protein	2155	2153
Ligand/ion	1 Zn, 1 GOL†, 2 CO_2_	1 Zn, 1 GOL, 1 CO_2_
Water	429	441
*B* factors (Å^2^)		
Protein		
Main chain	7.9	9.5
Side chain	11.2	12.8
Ligand/ion	3.6 (Zn), 12.6 (GOL), 9.5 (first CO_2_), 36.4 (second CO_2_)	4.9 (Zn), 15.2 (GOL), 15.2 (CO_2_)
Water	29.3	28.8
R.m.s. deviations		
Bond lengths (Å)	0.023	0.024
Bond angles (°)	2.223	2.206

† Glycerol.
