# Supplementary material for: Active-site solvent replenishment observed during human carbonic anhydrase II catalysis
Source: IUCrJ. 2018 Jan 1;5(Pt 1):93–102. doi: 10.1107/S2052252517017626 (PMC5755581; doi:10.1107/S2052252517017626)
Supplement: Supplementary file 1 [file m-05-00093-sup1.pdf]

IUCrJ

Volume 5 (2018)

Supporting information for article:

Active-site solvent replenishment observed during human carbonic anhydrase II catalysis

Jin Kyun Kim, Carrie L. Lomelino, Balendu Sankara Avvaru, Brian P. Mahon, Robert McKenna, SangYoun Park and Chae Un Kim

S1. Structural analysis on the bound water molecules

To compare the bound water molecules in the active site and the entrance conduit, we carefully refined water molecules in the two new structures (5Y2S for 7.0 atm CO_2 hCA II, and 5R2R for 2.5 atm CO_2 hCA II) and in the previous three structures (5DSI for 15 atm CO_2 hCA II, 5DSJ for 15 atm CO_2 hCA II – 50s, 5DSN for CO_2 hCA II – 1h) based on the PDB and COOT validation checks and the electron density maps (cutoff level of 1σ in $2F_o$-F_c electron density map). The re-refined previous structures were updated in the PDB with new PDB codes of 5YUI (redirected from 5DSI), 5YUJ (redirected from 5DSJ), and 5YUK (redirected from 5DSN). The important bound water molecules addressed in the main manuscript are listed in Table S1 to guide readers.

We have tested the consistency and reproducibility of the bound water molecules in the active site and the entrance conduit carefully (detailed data not shown). It was found that the different measuring conditions, such as data collection with detector offset as described in section 2.4 of the main manuscript, has negligible effects on the observed bound water molecules. We have also compared the ultra-high resolution structures of 7.0 atm CO_2 hCA II and 2.5 atm CO_2 hCA II with the structures refined with lower resolution cutoffs at 1.25 Å and 1.45 Å, respectively. It was found that the structures of 7.0 atm CO_2 hCA II and 2.5 atm CO_2 hCA II preserve all the key features in the bound water molecules, regardless of the diffraction resolution (at least up to 1.45 Å), $I/\sigma(I)$, completeness, and redundancy. These results suggest that the observed bound water molecules are the consequence of different CO_2 internal pressure levels, rather than the different measuring conditions or data analysis cutoffs.

There are several closely positioned water molecules in the active site and the entrance conduit of the five hCA II structures. Since most of these waters are transiently existing, it is expected that they can be located closer than the normal stably bound water molecules. In this regards, closely distanced water molecules near the active site and entrance conduit regions were also included in the final coordinates.

Finally, it should be noted that the five crystal structures described in our study were determined at a cryogenic temperature (100 K). Care needs to be taken when the cryo-crystallographic structures are related to the proteins' biological functions. In the case of hCA II, it is known that the protein remains biologically active in a crystalline state. In addition, our previous cryo-crystallographic studies on hCA II revealed biologically relevant intermediate states of hCA II (Kim *et al*., 2016). In this regards, it is assumed in the current study that the observed cryo-crystallographic structures reflect the snapshots of the biologically relevant intermediate states of hCA II. Future studies, such as using neutron crystallography or X-ray free electron lasers, may reveal more details on the bound water molecules in a biologically functional state of hCA II.

Table S1 List of key water molecules in the five hCA II structures.

	**15 atm (5YUI – redirected from 5DSI)**	**7.0 atm (5Y2S)**	**2.5 atm (5Y2R)**	**15 atm-50s (5YUJ – redirected from 5DSJ)**	**15 atm–1h (5YUK – redirected from 5DSN)**
$\mathbf{W_{DW}}$	-	-	-	A 626	A 584
$\mathbf{W_{DW}'}$	-	-	-	A 629	A 559
$\mathbf{W_{Zn}}$	A 454	A 455	A 486	A 431	A 437
$\mathbf{W_{I}}$	A 537	A 457	A 561	-	-
$\mathbf{W_{I}'}$	A 659	A 669	A 708	A 484	-
**W1**	-	A 595	A 619	A 557	A 530
**W2**	A 698	A 728	A 744	A 652	A 603
**W2'**	A 401	A 402	A 403	A 401	-
**W3a**	A 550	A 551	A 565	A 528	A 496
**W3b**	A 472	A 449	A 475	A 470	A 473
**W3b'**	A 702	A 729	-	-	-
$\mathbf{W_{EC1}}$	A 667	A 691	A 713	A 635	A 613
$\mathbf{W_{EC1}'}$	A 718	A 738	-	-	-
$\mathbf{W_{EC1}''}$	A 676	-	A 652	-	-
$\mathbf{W_{EC2}}$	-	A 678	A 706	A 609	A 588
$\mathbf{W_{EC2}'}$	A 690	A 718	A 732	A 653	-
$\mathbf{W_{EC3}}$	A 736	A 765	A 772	A 678	A 643
$\mathbf{W_{EC4}}$	A 773	A 815	A 824	A 698	A 661
$\mathbf{W_{EC5}}$	A 428	A 423	A 436	A 422	A 431
$\mathbf{W_{EC5}'}$	-	A 656	A 696	-	-

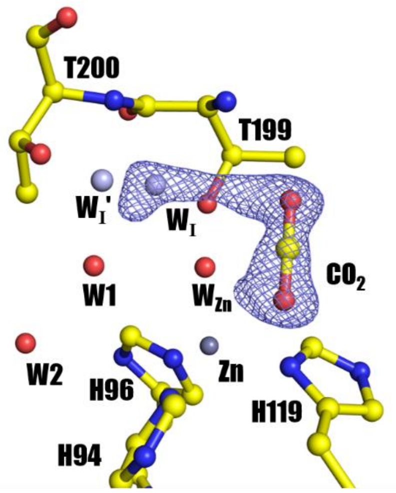

Figure S1 The active site of 2.5 atm CO_2 hCA II. The electron density $2F_o$-F_c map is contoured at 0.8σ, which shows the electron density extension from W_I to the CO_2 molecule.

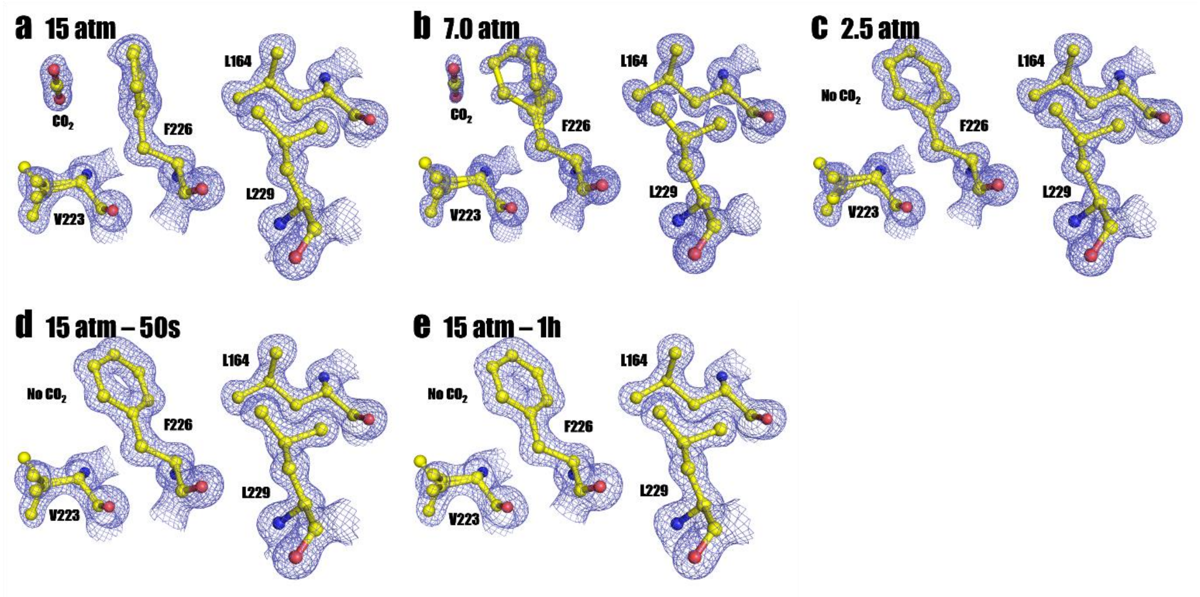

Figure S2 Secondary CO_2 binding sites of hCA II at different internal CO_2 pressures. (a) 15 atm CO_2 hCA II, (b) 7.0 atm CO_2 hCA II, (c) 2.5 atm CO_2 hCA II, (d)15 atm CO_2 hCA II – 50s, (e) 15 atm CO_2 hCA II – 1h. In all cases, the electron density $2F_o$-F_c maps (in *blue*) are contoured at 1.5σ. Although the secondary CO_2 binding is clearly visible in (a), it starts to fade out in (b). In the remaining internal CO_2 pressures, the secondary CO_2 completely disappears (c-e). Note that the side chain F226 rotates to accommodate the secondary CO_2 in (a), and that it appears with dual conformations in the lower internal CO_2 pressure in (b).

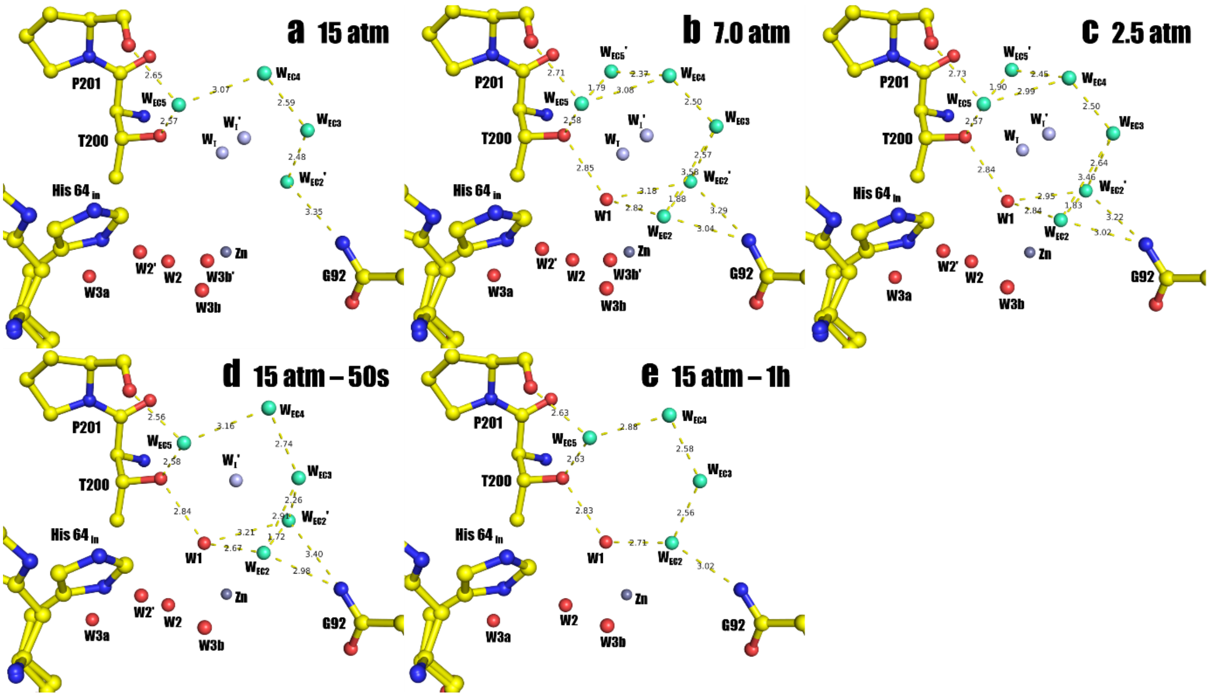

Figure S3 The hydrogen bonding network stabilizing the entrance conduit water molecules in (a) 15 atm CO_2 hCA II, (b) 7.0 atm CO_2 hCA II, (c) 2.5 atm CO_2 hCA II, (d) 15 atm CO_2 hCA II – 50s, (e) 15 atm CO_2 hCA II – 1h. The intermediate waters W_I and W_I' are colored in light gray and the entrance conduit waters are colored in cyan for clarity. Elaborate hydrogen bonding network exists between waters of W1, W_{EC1} (not shown in this figure view), W_{EC2}, W_{EC3}, W_{EC4}, W_{EC5}, W_I, and W_I' and also the three residues (G92, T200, and P201) of hCA II. Here, distances are shown only for the hydrogen bonding interactions that are mediated by waters of W1, W_{EC2}, W_{EC3}, W_{EC4}, W_{EC5} and the residues of G92, T200, and P201. Note the appearance of W1 starting from 7.0 atm CO_2 hCA II (b), and the complete disappearance of W_I and W_I' in 15 atm CO_2 hCA II – 1h (e).

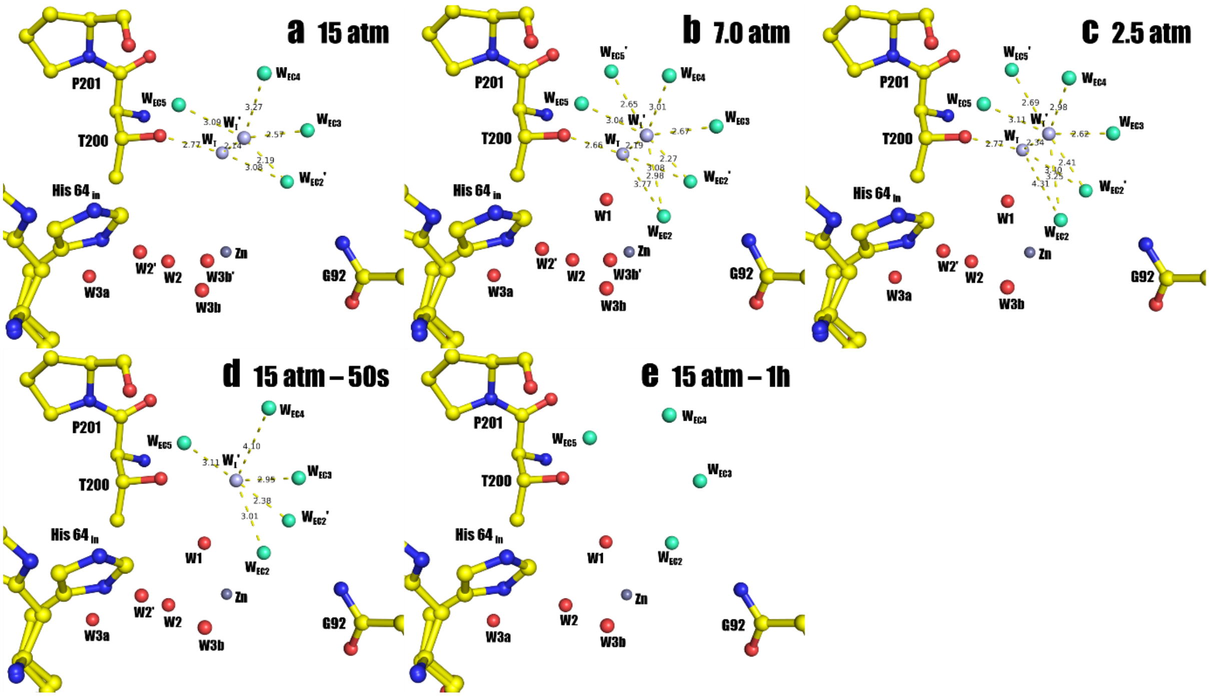

Figure S4 The hydrogen bonding network stabilizing the intermediate waters W_I and W_I' in (a) 15 atm CO_2 hCA II, (b) 7.0 atm CO_2 hCA II, (c) 2.5 atm CO_2 hCA II, (d) 15 atm CO_2 hCA II – 50s, (e) 15 atm CO_2 hCA II – 1h. The intermediate waters W_I and W_I' are colored in light gray and the entrance conduit waters are colored in cyan for clarity. Elaborate hydrogen bonding network exists between W_I/W_I' and W_{EC2}, W_{EC3}, W_{EC4}, and W_{EC5} for their stabilization. Here, distances are shown only for the hydrogen bonding interactions that are mediated *via* W_I and W_I'. Hence, no distance information is included for 15 atm CO_2 hCA II – 1h (e) where complete disappearance of W_I and W_I' has happened.

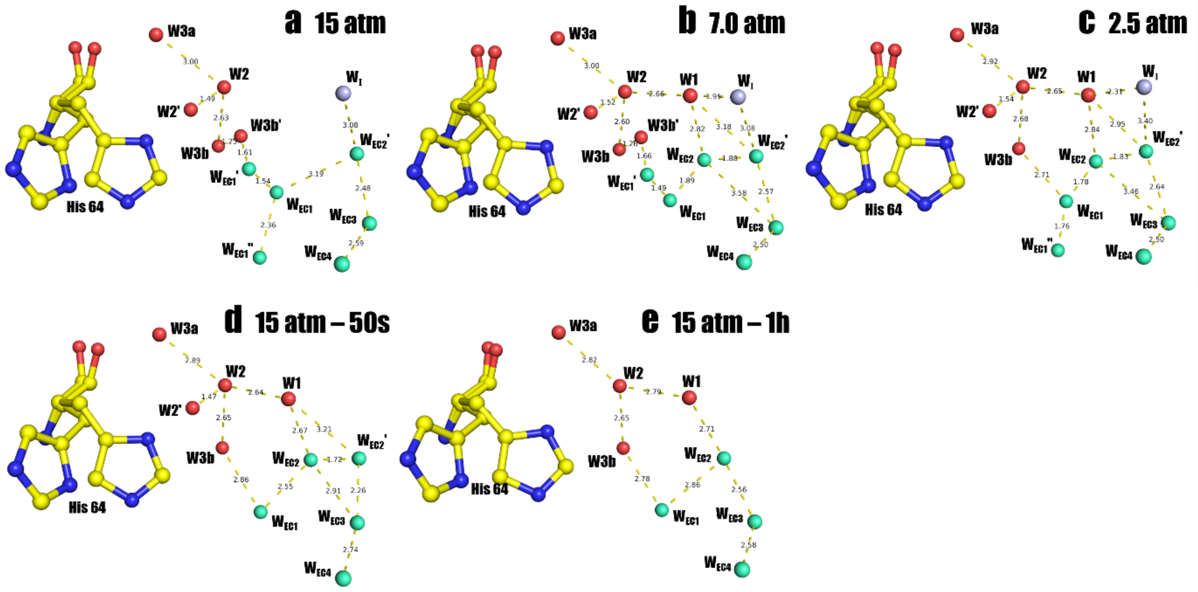

Figure S5 The distance information between the active site waters and the entrance conduit waters observed in (a) 15 atm CO_2 hCA II, (b) 7.0 atm CO_2 hCA II, (c) 2.5 atm CO_2 hCA II, (d) 15 atm CO_2 hCA II – 50s, (e) 15 atm CO_2 hCA II – 1h. The intermediate waters W_I and W_I' are colored in light gray and the entrance conduit waters are colored in cyan for clarity. Elaborate hydrogen bonding network (distance range from 2.2 Å to 4.0 Å) exists between waters mediating proton transfer (W1/W2/W2′/W3a/W3b/W3b′), intermediate waters (W_I/W_I'), and the entrance conduit waters (W_{EC1}, W_{EC2}, W_{EC3}, W_{EC4}, and W_{EC5})
